# Phytoplankton growth and stoichiometric responses to warming, nutrient addition and grazing depend on lake productivity and cell size

**DOI:** 10.1111/gcb.14660

**Published:** 2019-06-01

**Authors:** Marika A. Schulhof, Jonathan B. Shurin, Steven A. J. Declerck, Dedmer B. Van de Waal

**Affiliations:** ^1^ Division of Biological Sciences, Section of Ecology, Behavior & Evolution University of California San Diego La Jolla California; ^2^ Department of Aquatic Ecology, Netherlands Institute of Ecology (NIOO‐KNAW) Wageningen the Netherlands

**Keywords:** communities, ecological stoichiometry, eutrophication, grazing, multiple stressors, phytoplankton, productivity, warming

## Abstract

Global change involves shifts in multiple environmental factors that act in concert to shape ecological systems in ways that depend on local biotic and abiotic conditions. Little is known about the effects of combined global change stressors on phytoplankton communities, and particularly how these are mediated by distinct community properties such as productivity, grazing pressure and size distribution. Here, we tested for the effects of warming and eutrophication on phytoplankton net growth rate and C:N:P stoichiometry in two phytoplankton cell size fractions (<30 µm and >30 µm) in the presence and absence of grazing in microcosm experiments. Because effects may also depend on lake productivity, we used phytoplankton communities from three Dutch lakes spanning a trophic gradient. We measured the response of each community to multifactorial combinations of temperature, nutrient, and grazing treatments and found that nutrients elevated net growth rates and reduced carbon:nutrient ratios of all three phytoplankton communities. Warming effects on growth and stoichiometry depended on nutrient supply and lake productivity, with enhanced growth in the most productive community dominated by cyanobacteria, and strongest stoichiometric responses in the most oligotrophic community at ambient nutrient levels. Grazing effects were also most evident in the most oligotrophic community, with reduced net growth rates and phytoplankton C:P stoichiometry that suggests consumer‐driven nutrient recycling. Our experiments indicate that stoichiometric responses to warming and interactions with nutrient addition and grazing are not universal but depend on lake productivity and cell size distribution.

## Graphical abstract

We measured the response of three phytoplankton communities to multifactorial combinations of temperature, nutrient and grazing treatments. Nutrients elevated net growth rates and reduced carbon:nutrient and nitrogen:phosphorus ratios of all communities. Warming effects on growth and stoichiometry depended on lake productivity: warming enhanced growth in the most productive community and caused strongest stoichiometric responses in the least productive community. Grazing reduced C:P and N:P ratios in the least productive community, suggesting consumer‐driven nutrient recycling. Our experiments indicate that stoichiometric responses to warming, and interactions with nutrient supply and grazing, depend on lake productivity and cell size distribution.

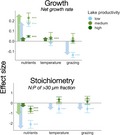

## 1 INTRODUCTION

Global environmental change is currently shifting nutrient fluxes and climate in ways that affect the structure and functioning of food webs. Changes in nutrient supply due to eutrophication and climate warming may alter the elemental composition of primary producers with consequences for higher trophic levels (De Senerpont Domis, Van de Waal, Helmsing, Van Donk, & Mooij, 2014; Van de Waal, Verschoor, Verspagen, Van Donk, & Huisman, 2010). Surface temperatures of lakes worldwide have warmed significantly since 1985, and in some areas, have increased more rapidly than air temperature (O'Reilly et al., 2015; Schneider & Hook, 2010). Additionally, point and nonpoint sources of nutrients to water bodies cause eutrophication due to excessive phosphorus (P) and nitrogen (N) inputs (Carpenter et al., 1998). Human enrichment of terrestrial and aquatic systems with N and P also changes biogeochemical cycling processes and results in stoichiometrically imbalanced systems and altered nutrient limitation patterns with attendant consequences for food webs (Elser, Kyle, Steuer, Nydick, & Baron, 2009; Sickman, Melack, & Clow, 2003; Sterner & Elser, 2002; Van de Waal et al., 2010).

Ecological stoichiometry describes the balance of energy (as carbon [C]) and nutrients between organisms and their environment and can inform our understanding of the impacts of environmental change on food webs (Hessen, Elser, Sterner, & Urabe, 2013; Sterner & Elser, 2002; Van de Waal, Elser, Martiny, Sterner, & Cotner, 2018). Elemental ratios of autotrophic biomass are important for understanding relationships between environmental nutrient supply, uptake by autotrophs, species composition, producer–consumer interactions and biogeochemical cycling. Elemental stoichiometry of phytoplankton, the predominant autotrophs in many aquatic systems, is primarily influenced by environmental supply of inorganic resources, most notably N, P and light (Sterner & Elser, 2002). Moreover, phytoplankton stoichiometry depends on various traits, such as cell size and growth rate (reviewed in Finkel et al., 2010).

Cell size of phytoplankton influences key cellular processes such as nutrient uptake and utilization strategies, in addition to trophic interactions. Allometric scaling relationships show that small cells tend to have higher maximum growth rates and acquire limiting nutrients more efficiently due to the high surface area to volume ratio and smaller diffusion boundary layer, whereas large cells have greater maximum uptake rates per cell and may have larger internal nutrient storage capacity (reviewed in Litchman & Klausmeier, 2008; Litchman, Klausmeier, Schofield, & Falkowski, 2007). Low‐nutrient environments should therefore favor small cells that are strong nutrient competitors, whereas high and fluctuating nutrient environments should be dominated by large‐celled species (Cloern, 2018; Edwards, Klausmeier, & Litchman, 2011; Irwin, Finkel, Schofield, & Falkowski, 2006; Litchman et al., 2007; Litchman, de Tezanos Pinto, Klausmeier, Thomas, & Yoshiyama, 2010). In addition, the size of phytoplankton affects trophic interactions and susceptibility to grazing by zooplankton, as increasing cell size results in greater resistance to gape‐limited grazers (reviewed in Litchman et al., 2010; Litchman & Klausmeier, 2008; Ward, Dutkiewicz, & Follows, 2014).

Elemental ratios provide insights into how resources are allocated within cells to support cellular functions and overall metabolism. For instance, investment in P‐rich ribosomes is required for growth, whereas N‐rich proteins are required for resource acquisition. The growth rate hypothesis posits that allocation of P to ribosomes increases as growth rates increase, resulting in reduced N:P ratios (Elser et al., 2003; but see Flynn et al., 2010). Optimal N:P ratios vary across phytoplankton taxa and reflect nutrient requirements determined by their cellular machinery (Klausmeier, Litchman, Daufresne, & Levin, 2004), as well as the degree of stoichiometric plasticity, which is influenced by nutrient storage capacity (Hall, Smith, Lytle, & Leibold, 2005). Phytoplankton N:P ratios are less variable at high growth rates when nutrients are not limiting, but vary substantially under nutrient limitation (Hillebrand et al., 2013). However, phytoplankton N:P ratios have also been shown to vary widely within a population of the same species under unconstrained growth conditions (Brandenburg et al., 2018), indicating that N:P ratios are regulated genetically as well as by a suite of biotic and abiotic factors.

Eutrophication is expected to reduce carbon:nutrient ratios of phytoplankton due to increased nutrient availability and light limitation (Dickman, Vanni, & Horgan, 2006; Sterner & Elser, 2002), while N:P ratios depend primarily on the supplies of N and P. The effects of warming on shifts in phytoplankton stoichiometry, however, are less understood. Warming can increase N:P in eukaryotic phytoplankton due to increased rates of protein synthesis and a reduction in the quantity of ribosomes required to produce proteins (Toseland et al., 2013) or shifts in community composition toward species with lower P demands (Yvon‐Durocher, Schaum, & Trimmer, 2017). Warming has also been shown to increase C:nutrient stoichiometry of a phytoplankton community, particularly under oligotrophic (low P) conditions, likely due to enhanced nutrient use efficiencies (De Senerpont Domis et al., 2014). However, warming has also been found to reduce C:P and N:P ratios, presumably caused by increased nutrient availability as a result of nutrient recycling by consumers or heterotrophic microbes (Velthuis et al., 2017). Grazing can alter phytoplankton stoichiometry due to consumer‐driven nutrient recycling, which increases nutrient availability for phytoplankton (Elser & Urabe, 1999). Conversely, as zooplankton tend to have higher nutrient demands and exhibit lower stoichiometric plasticity compared with phytoplankton (reviewed in Meunier et al., 2017), they can also increase nutrient limitation for phytoplankton by sequestering limiting nutrients in their tissues (Elser & Urabe, 1999). The effects of warming on phytoplankton stoichiometry are thus likely to interact with nutrient loading as well as the abundance of zooplankton.

Here, we tested the effects of warming, eutrophication and grazing on phytoplankton growth and stoichiometry in multifactorial experiments on phytoplankton communities from three Dutch lakes distributed across a productivity gradient. We measured net growth rates, N:P and C:P for two size fractions (<30 µm and >30 µm) for all three communities, and tested for the independent and interactive effects of nutrient addition, warming and grazing. We expected that C:P and N:P would decrease as a result of nutrient addition, and that the effect of grazing would vary by lake, due to differences in phytoplankton size structure and thus edibility by grazers (i.e. *Daphnia*). Specifically, we anticipated strongest grazing effects in communities from the lowest productivity system, as we expect these to be dominated by smaller, more edible phytoplankton. Lastly, we hypothesized that the effect of warming on stoichiometry may vary by community and interact with nutrient supply, such that under low nutrient conditions, warming will constrain phytoplankton growth and lead to enhanced accumulation of excess elements. Our goal was to understand whether climate warming and eutrophication exert consistent independent or interactive effects on phytoplankton stoichiometry, or whether their effects depend on lake trophic status, cell size, or the presence of zooplankton grazers.

## 2 MATERIALS AND METHODS

### 2.1 Field sampling and experimental setup

Spring phytoplankton communities were collected from three lakes differing in trophic status, sampled 1 month apart: Maarsseveen (52.144402N, 5.080691E; March 2017), Tjeukemeer (52.890225N, 5.802871E; April 2017) and Loosdrecht (52.196582N, 5.080495E; May 2017). The community from Maarsseveen was comprised primarily of small flagellated green algae, diatoms (*Aulacoseira*, *Asterionella*) and mucilaginous cyanobacterial colonies. Tjeukemeer was dominated by filamentous cyanobacteria, but also contained medium‐sized green algae (*Scenedesmus*, *Pediastrum*), pennate diatoms and mucilaginous cyanobacterial colonies. Loosdrecht was also dominated by filamentous cyanobacteria, as well as by mucilaginous cyanobacterial colonies, and small‐sized green algae such as *Scenedesmus*. At each lake, 340 L of water from 0.5 to 1.0 m depth was collected in 10 L containers and brought back to the laboratory to inoculate experiments. Additionally, depth profiles of water temperature and pH were recorded using HydroLab sensors (OTT Hydromet, USA), and water samples were collected for dissolved nutrient analyses (DIN, DIP) and seston samples were collected onto prerinsed glass microfiber filters (Whatman GF/F, Maidstone, UK) in triplicate for chlorophyll‐*a* (chl‐*a*) analysis and C, N, P elemental analysis. Plankton inocula were stored in the laboratory in the dark overnight and experiments were inoculated the next morning. All inocula were prescreened through a 200 µm mesh to remove large zooplankton grazers, and gently mixed in a large cattle tank before filling equal 10 L volumes into transparent Nalgene containers.

Using a full factorial design, the culture containers were subjected to two temperature, nutrient and grazing treatments, for a total of eight factorial treatment combinations. Each of the eight treatments was replicated four times, resulting in 32 experimental units for each of three experiments. The temperature treatments consisted of an ambient treatment set to the lake water temperature at the time of sampling, and a +4°C warming treatment based on plausible global change scenarios (IPCC scenario RCP8.5, Pachauri et al., 2014). However, due to technical problems with temperature control in the incubation system, there were differences between the magnitude of warming for each experiment. The resulting mean ambient and elevated temperatures, respectively, for each experiment were as follows: 9.6 ± 0.5 and 11.0 ± 0.2°C for Maarsseveen, 12.0 ± 0.4 and 15.0 ± 0.5°C for Tjeukemeer and 15.8 ± 0.3 and 20.0 ± 0.2 for Loosdrecht.

For the nutrient addition treatment, nitrogen and phosphorus were added in an N:P molar ratio of 16:1 (1 mM NO_3_^−^ and 0.0625 mM PO_4_^3−^) corresponding to the Redfield ratio (Redfield, 1934, 1958). For the grazing treatment, *Daphnia* was added to a final population density of five *Daphnia magna* individuals per liter. *Daphnia* were acquired commercially (Ruinemans Aquarium B.V., Montfoort, the Netherlands), and individuals were selected, cleaned and subsequently cultured in the laboratory fed with *Chlamydomonas reinhardtii* cultures at 0.5 mg C L^−1^ day^−1^. For each experiment, adult individuals of a standardized size were selected and thoroughly washed in deionized water before being added to culture vessels. Culture vessels were randomly positioned and submerged in temperature‐controlled aquaria using the Farex SR minisystem (RKC Instruments, Tokyo, Japan) and subjected to controlled light conditions (120 µmol photons m^−2^ s^−1^) with a day–night cycle of 14:10 that simulated the spring light conditions in the Netherlands. Every 2 days, chl‐*a* samples were collected from each culture vessel by gentle mixing and using a depth‐integrated tube sampler. Chl‐*a* concentrations were quantified using a Phyto‐PAM fluorometer (Walz, Germany). Each experiment ran for a duration of 6 days, when phytoplankton communities started to enter the stationary phase of growth.

The experiments were harvested on day 6 when samples from each culture vessel were collected for the analysis of chl‐*a*, particulate C, N, P, dissolved inorganic N and P, flow cytometry and microscopy. Additionally, temperature and pH inside of each culture vessel were recorded. For chl‐*a* analyses, samples were analyzed in two ways: fluorometrically (Phyto‐PAM, Walz, Germany) and using high‐performance liquid chromatography (HPLC). For the chl‐*a* (HPLC) and elemental analyses, seston samples were filtered onto prerinsed glass microfiber filters (Whatman GF/F, Maidstone, UK) in two size fractions: for the whole community and <30 µm fraction (separated using 30 µm mesh). Molar elemental quantities for the smaller size fraction were subtracted from the whole fraction to calculate molar ratios for the >30 µm size fraction.

Samples for chl‐*a* were collected on GF/F filters (Whatman) and stored in Eppendorf tubes at −20°C. Prior to extraction, filters were thawed for 30 min at room temperature, and 1.5 ml of 80% ethanol was added. The tubes were subsequently placed in a water bath at 80°C for 10 min in the dark. After manual mixing, 1 ml of the sample was syringe filtered (0.45 µm) and immediately analyzed on an HPLC UltiMate 3000 (Thermo Scientific) equipped with a Hypersil ODS column (25 cm, 5 µm, 4.6 × 250 mm; Agilent) and an RF 2000 fluorescence detector (Dionex/Thermo Scientific).

Filtrate samples were collected in polyethylene containers and stored at −20°C for analyses of dissolved inorganic nitrogen (DIN, including NO_3_^−^, NO_2_^−^ and NH_4_^+^) and phosphorus (including soluble reactive phosphate SRP), and seston samples on filters were dried at 60°C for 24 hr and stored in a desiccator until further analysis. SRP was determined by absorption at 715 nm following Murphy and Riley (1962). Ammonium (NH_4_^+^), nitrite (NO_2_^−^) and total oxidized nitrogen (NO_2_^−^ + NO_3_^−^) were determined using a Technicon TV AAcs 800 autoanalyzer (Technicon, Tarrytown, New York), and NO_3_^−^ was obtained through subtraction of nitrite from total oxidized nitrogen. P content in seston retentate was assessed by incinerating the samples for 30 min at 500°C, followed by a 2% persulphate digestion step in the autoclave for 30 min at 121°C. The digested samples were analyzed using a QuAAtro segmented flow analyzer (Seal Analytical Incorporated, Beun de Ronde, Abcoude, the Netherlands). C and N content in seston retentate was determined using a FLASH 2000 organic elemental analyzer (Brechbueler Incorporated, Interscience B.V., Breda, the Netherlands).

### 2.2 Data analysis

All statistical analyses were performed using the statistical program R version 3.4.2 (R Core Team, 2017). We tested for a productivity gradient in the initial field data from lakes by testing for differences in the mean values of TN (µm), TP (µm) and chl‐*a* (µg/L) measured in triplicate in the three lakes using one‐way ANOVA and Tukey's posthoc test. Net growth rates in each treatment were calculated by dividing the difference between the ln‐transformed chl‐*a* values from the beginning and end of the experiment by the duration of the experiment: [ln(chl‐*a*_day6_) − ln(chl‐*a*_day0_)]/6.

We fit generalized linear models (“glm” function in lme4 package) with Gaussian distributions to determine the main and interactive effects, and effect sizes (parameter estimates from models; see below), of experimental treatments on response variables (net growth rate, N:P and C:P <30 µm and N:P and C:P >30 µm, dissolved nutrients) in each experiment and assessed statistical significance using a chi‐squared test. Stoichiometric (N:P and C:P <30 µm; N:P and C:P >30 µm) and dissolved nutrient data were ln‐transformed prior to running generalized linear models. Parameter estimates and standard error values from the models were used to represent effect sizes of treatments on response variables. We also tested for differences between size fractions for ln‐transformed N:P and C:P in control treatments (ambient nutrient and temperature conditions, without added grazers) using one‐way ANOVA and Tukey‧s post‐hoc test.

## 3 RESULTS

### 3.1 Lake data

**Figure 1:**
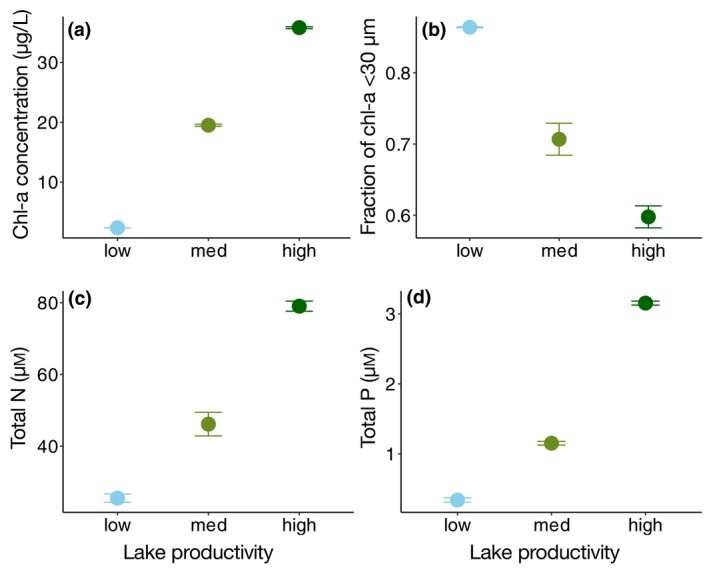
Overview of the lake communities with (a) total chl‐*a* concentration, (b) fraction of chl‐*a* in the <30 µm size fraction, (c) total nitrogen (TN) and (d) total phosphorus (TP) across a lake productivity gradient. Values denote mean ± *SE* (*n* = 3)

Mean chl‐*a*, fraction of chl‐*a* <30 µm, TN (µm) and TP μM were significantly different among the three sampled lakes (*p* < 0.01; Table 1). Highest chl‐*a* concentrations occurred in Lake Tjeukemeer (35.8 ± 0.2 µg/L), followed by Lake Loosdrecht (19.5 ± 0.2 µg/L) and Lake Maarsseveen (2.37 ± 0.01 µg/L; Figure 1a). The fraction of chl‐*a* <30 µm showed the opposite pattern: the lowest fraction occurred in Tjeukemeer (0.60 ± 0.02), followed by Loosdrecht (0.71 ± 0.02) and Lake Maarsseveen (0.86 ± 0.001; Figure 1b). Similar to mean chl‐*a*, TN and TP were highest in Lake Tjeukemeer followed by Loosdrecht, whereas the lowest concentrations were observed in Lake Maarsseveen (Figure 1c,d; Table 1). Therefore, phytoplankton communities from lakes Maarsseveen, Loosdrecht, Tjeukemeer will be referred to as the “low,” “medium” and “high” productivity communities, respectively. Dissolved inorganic phosphorus (DIP) concentrations averaged 0.53 µm for Lake Tjeukemeer, and below detection (i.e. <0.01 µm) for Loosdrecht and Maarsseveen. All three lakes did still contain detectable concentrations of DIN, with 9.9 µm, 2.8 µm, and 15.9 µm, for Lake Tjeukemeer, Loosdrecht and Maarsseveen, respectively (Table 1).

**Table 1:** Lake chemistry at the time of sampling with concentrations (µm) of total N (TN) and P (TP), particulate inorganic N (PON) and P (POP) and dissolved inorganic N (DIN) and P (DIP), and their ratios

Productivity	TN	TP	TN:TP	PON	POP	PON:POP	DIN	DIP	DIN:DIP
Low	25.5** ± **1.2	0.34** ± **0.03	77.2 ± 11.7	9.6** ± **1.2	0.34** ± **0.03	29.5** ± **6.8	15.9	<0.01	—
Medium	46.2 ± 3.3	1.15** ± **0.03	40.2 ± 3.4	43.4 ± 3.3	1.15** ± **0.03	37.8** ± **3.4	2.8	<0.01	—
High	79.1 ± 1.4	3.15** ± **0.03	25.1 ± 0.6	69.2** ± **1.4	2.63** ± **0.03	26.3** ± **0.7	9.9	0.53	18.8

Note

Values denote mean ± *SE* (*n* = 3; *n* = 1 for DIN and DIP).

### 3.2 Net growth rate

**Figure 2:**
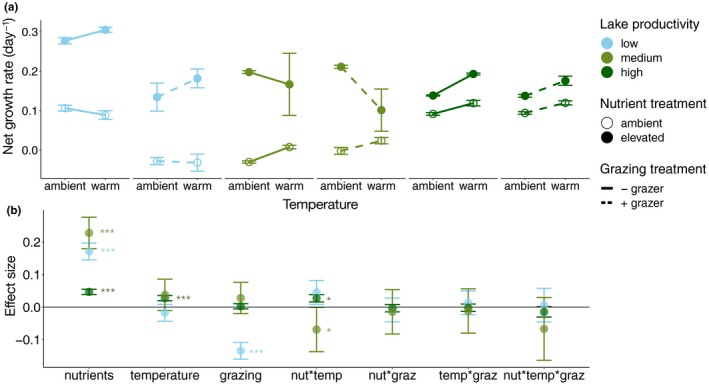
Net community growth rates in experimental treatments across a productivity gradient with (a) mean values ± *SE* (*n* = 4) for each combination of temperature, grazing and nutrient treatments, and (b) effect sizes ± *SE* from generalized linear models for each community, with asterisks indicating significance values (*p < 0.05, **p < 0.01, ***p < 0.001)

Nutrient addition significantly increased net growth rates of all three communities (*p* < 0.001, Figure 2a), whereas the effects of temperature and grazing varied by community. Nutrients increased net growth rate most for the medium and low productivity communities. The low productivity community was the only one that experienced reduced net growth rates as a result of grazing, across all temperature and nutrient treatments (*p* < 0.001, Figure 2a). In the high productivity community, warming alone elevated net growth rates (*p* < 0.001, Figure 2a), and in the medium and high productivity communities, temperature and nutrients interacted significantly (*p* < 0.05, Figure 2b). However, the direction of the interactive effect differed by community. In the medium productivity community, warming had a positive effect on net growth rate at ambient nutrient levels, but at elevated nutrient levels, the effect of warming was negative (*p* < 0.05, Figure 2a). The opposite result, a positive nutrient × warming interaction, was found in the most eutrophic community where nutrient addition stimulated higher net growth rates at high temperature (*p* < 0.05, Figure 2a).

### 3.3 Stoichiometry

**Figure 3:**
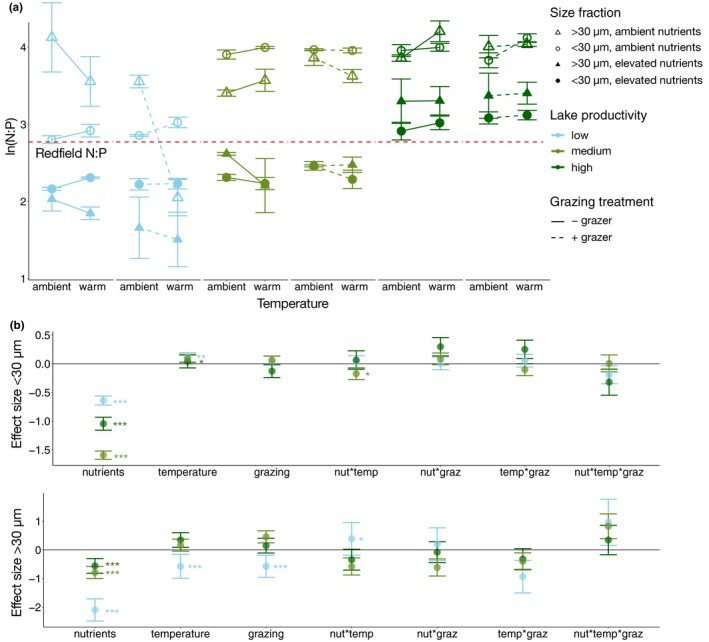
Natural log‐transformed N:P in experimental treatments across a productivity gradient for two cell size fractions (<30 µm and >30 µm) with (a) mean values ± *SE* (*n* = 4) for each combination of temperature, grazing and nutrient treatments, and (b) effect sizes ± *SE* from generalized linear models for each community and size fraction, with asterisks indicating significance values (*p < 0.05, **p < 0.01, ***p < 0.001). The dashed horizontal line shows the ln‐transformed Redfield N:P (16:1) for reference

Phytoplankton N:P ratios varied among communities and size classes in response to treatments. In ambient conditions (ambient temperature and nutrient levels without added grazers), N:P ratios differed between the <30 µm and >30 µm size fractions for two communities. In the low productivity community, N:P in the larger size fraction was significantly higher than in the smaller size fraction (*p* < 0.05, Figure 3a), whereas in the medium productivity system, the opposite pattern occurred (*p* < 0.001, Figure 3a). There were no significant differences in N:P for the two size fractions at ambient conditions for the high productivity community (Figure 3a).

Nutrient addition reduced N:P for all three communities and both size fractions (*p* < 0.001, Figure 3a), with the largest effect size on the smaller size fraction in the medium productivity community and on the larger size fraction in the low productivity community (Figure 3b). Warming had variable effects on N:P depending on the size fraction and community. In the low productivity community, for instance, warming increased N:P in the smaller fraction (*p* < 0.01, Figure 3a), whereas it decreased N:P in the larger fraction (*p* < 0.001, Figure 3a). The magnitude of warming effects generally became smaller when nutrients were added (temperature × nutrient interaction, *p* < 0.05, Figure 3a). Grazing also caused a reduction in N:P, but only in the larger size fraction of the low productivity community (*p* < 0.001, Figure 3a).

**Figure 4:**
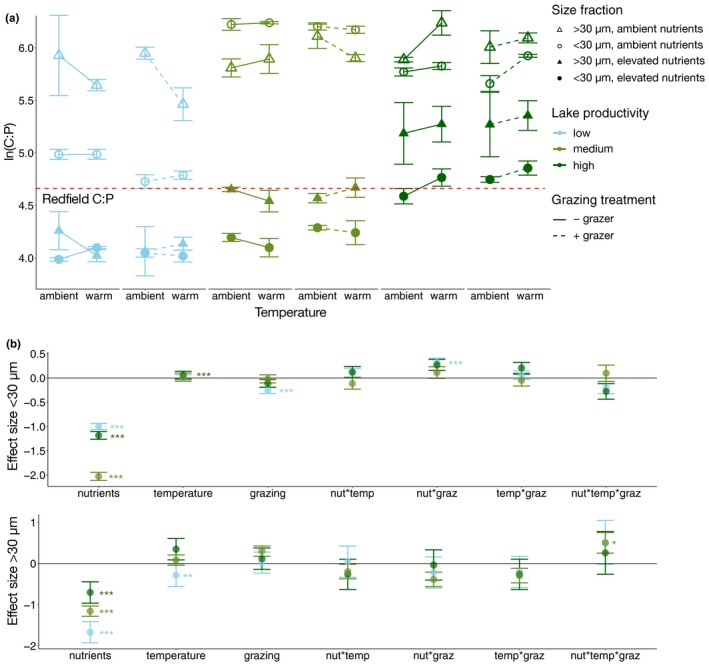
Natural log‐transformed C:P in experimental treatments across a productivity gradient for two cell size fractions (<30 µm and >30 µm) with (a) mean values ± *SE* (*n* = 4) for each combination of temperature, grazing and nutrient treatments, and (b) effect sizes ± *SE* from generalized linear models for each community and size fraction, with asterisks indicating significance values (*p < 0.05, **p < 0.01, ***p < 0.001). The dashed horizontal line shows the ln‐transformed Redfield C:P (106:1) for reference

Similar to N:P, phytoplankton C:P ratios varied in response to the treatments among communities and size classes. Phytoplankton C:P ratios in ambient conditions (ambient nutrients, temperature and without grazers) were higher in the larger size fraction as compared to the smaller size fraction for the highest productivity system (*p* < 0.05, Figure 4a), and marginally significant for the lowest productivity system (*p* = 0.05, Figure 4a). Conversely, in the medium productivity system, C:P ratios were higher in the smaller size fraction (*p* < 0.01, Figure 4a).

With nutrient addition, both size fractions show significant reductions in C:P for all three communities (*p* < 0.001, Figure 4a), with the largest effect size in the medium productivity community for the smaller size fraction and in the low productivity community for the larger size fraction (Figure 4b). Warming increased C:P in the smaller size fraction of the high productivity community (*p* < 0.001, Figure 4a) and decreased C:P in the larger size fraction of the low productivity community (*p* < 0.01, Figure 4a). Grazing at ambient nutrient levels lowered C:P in the smaller size fraction in the low productivity community (grazing × nutrient interaction, *p* < 0.001, Figure 4a). In the larger size fraction of the medium productivity community, there was a three‐way interaction between nutrients, warming and grazing, whereby grazing caused an increase in C:P at ambient temperature and nutrients, but a reduction in C:P when the same treatment was warmed. However, this effect was not observed when nutrients were added (*p* < 0.05, Figure 4a).

## 4 DISCUSSION

Our experiment showed that the effects of warming on phytoplankton growth and stoichiometry depended greatly on the supply of nutrients, the density of grazers, trophic status and cell sizes in each phytoplankton community. The only consistent effect across communities from three different lakes was that fertilization with N and P enhanced net growth rates and elevated the nutrient content of cells, with a decrease in N:P and C:P stoichiometry. Warming and grazers affected phytoplankton growth and stoichiometry, but the effects varied among communities, size classes and the interactions with other experimental treatments. Rampant interactions indicate that shifts in phytoplankton stoichiometry with lake warming are likely to occur but depend greatly on local environmental (biotic and abiotic) context. Projecting changes in lake food webs, nutrient cycles and global fluxes of C, N and P in response to global changes will therefore demand knowledge of local conditions and the many interactions involved in the control of phytoplankton stoichiometry.

### 4.1 Nutrient effects on growth

Nutrient addition strongly influenced net growth rates of all three communities. The low productivity community achieved the highest net growth rates under nutrient enrichment. This community was most strongly limited by nutrients and contained highest densities of smaller cells with high surface area to volume ratios that aid in acquiring nutrients (Litchman et al., 2007; Marañón et al., 2013) and also exhibit higher maximum growth rates (Banse, 1976; Litchman et al., 2007), possibly explaining the highest net growth rates in the low productivity community. The medium productivity community was dominated by filamentous cyanobacteria and had the lowest net growth rate in ambient conditions (ambient nutrients and temperature, without grazers; Figure 2a), consistent with findings that cyanobacteria have lower growth rates than diatoms (Edwards, Thomas, Klausmeier, & Litchman, 2012) that were more abundant in the low productivity community. Additionally, nutrients had the greatest effect on increasing net growth rates in this community (Figure 2b). Although the high productivity community was also dominated by filamentous cyanobacteria, its net growth rate at ambient conditions was similar to that of the low productivity community, while it exhibited the weakest response to nutrient addition. Even though the medium and high productivity communities were both dominated by cyanobacteria, their net growth rates at ambient conditions and the strength of their responses to nutrient addition varied, possibly due to differences in the strength of nutrient limitation or species composition between the two communities.

The strength of nutrient limitation, and therefore the responses to nutrient additions, can vary temporally depending on lake trophic status and the developmental phase of a bloom (Sommer et al., 2012). Because of the logistical necessity of conducting the three experiments in sequence, the three phytoplankton communities were at different points in their phenological trajectories at the time of the experiments. For example, experimental communities from the intermediate productivity lake were collected relatively late in the spring season (May). At this point in time, the community may have already reached its carrying capacity, which may explain our observations of relatively low net growth rates in the control treatments (ambient nutrient and temperature conditions, without addition of grazers) and stronger nutrient limitation, indicated by the stronger effect size of nutrient addition (Figure 2b). Although difficult to realize in practice, future comparative experimental studies on community responses to eutrophication, warming and grazing would benefit from a standardization of the timing of experiments in relation to the phase of phytoplankton community development across lakes.

### 4.2 Nutrient effects on stoichiometry

The stoichiometric responses to nutrient addition, warming and grazing were mediated by the size structure and associated taxonomic composition of the phytoplankton communities. We found significant differences in elemental ratios between the two size fractions which were more pronounced for C:P than N:P (Figures 3 and 4). The higher C:P ratios in the larger size fraction for the low and high productivity communities suggest that the larger cells were more nutrient‐limited than smaller cells, consistent with expectations for nutrient uptake traits associated with size (Litchman et al., 2007; Marañón et al., 2013). Moreover, the effects of each treatment on elemental ratios in the three lakes differed by size fraction, suggesting that traits associated with size, such as nutrient uptake efficiencies and grazer susceptibility, influence responses to each treatment. While C:P in the larger size fraction was most strongly affected by nutrient addition, showing a decrease with fertilization in all communities, C:P of the smaller size fraction also responded strongly to grazing, temperature, and their interactions. It is possible that smaller cells, with lower internal nutrient stores, responded more rapidly to perturbations in nutrient availability indirectly caused by warming and grazing.

Differences in stoichiometric responses to nutrient addition across the communities may have arisen due to variation in stoichiometric flexibility associated with the taxonomic composition and size structure of each community, as nutrient requirements for functional machinery are species‐specific (Klausmeier et al., 2004). Decreases in N:P and C:P ratios with increased nutrient supply indicate that the communities were primarily P‐limited and that phytoplankton rapidly take up P when it becomes more available. Moreover, these decreases in ratios may reflect increased P‐storage (reviewed in Meunier et al., 2017), or possibly increased growth rates following the growth rate hypothesis (Elser et al., 2003; but see Flynn et al., 2010). The effect size of nutrient addition on N:P and C:P differed by community and size fractions within communities, and may relate to the degree of nutrient limitation experienced by communities in their lake of origin. TN:TP ratios in lakes at the time when phytoplankton communities were collected were inversely related to the productivity of the three communities: TN:TP was highest in the lowest productivity lake, intermediate in the medium productivity community and lowest in the high productivity community, reflecting increasing P‐limitation from the high to low productivity systems (Table 1).

Fertilization resulted in changes in phytoplankton stoichiometry that depended on both cell size fraction and the productivity of the lake. For the smaller size fraction, nutrient addition had the greatest effect on C:P and N:P in the medium productivity system, followed by the high and low productivity systems, respectively (Figures 3b and 4b). However, in the larger size fraction, nutrient addition had the greatest effect on C:P and N:P in the low productivity community, followed by medium and high productivity, respectively (Figures 3b and 4b). This suggests that nutrient limitation and stoichiometric plasticity depend on community size structure and composition. Specifically, smaller cells in the medium productivity community and larger cells in the low productivity community appear to be the most P‐limited, and/or the most flexible in their stoichiometry, as they showed the strongest reductions in C:P and N:P in response to nutrient addition.

The smaller size fraction of the low productivity community had the lowest N:P and C:P of all three communities at ambient nutrient levels. This community consisted mostly of diatoms as well as fast‐growing, small‐celled phytoplankton species that are good nutrient competitors and tend to have both higher P content and a more constrained elemental composition (Elser et al., 2003; Martiny et al., 2013). Such traits might explain the weakest response to nutrient addition in the smaller size fraction of the low productivity community despite the lowest environmental P supply in the lake of origin. Therefore, the differences in the strengths of responses to nutrient additions may depend on differences in nutrient limitation, nutrient competitive abilities and stoichiometric flexibility between size fractions and across communities.

### 4.3 Warming effects on growth

Warming had a positive effect on net growth rate only in the high productivity community. These results are in line with previous studies indicating that the effect of warming on phytoplankton communities depends on trophic state and species composition, with more positive effects on growth in systems with high P supply (Elliott, Jones, & Thackeray, 2006; Huber, Adrian, & Gerten, 2008; Rigosi, Carey, Ibelings, & Brookes, 2014; Tadonléké, 2010). Additionally, the high productivity community was dominated by filamentous cyanobacteria, which tend to be favored under warm conditions (Kosten et al., 2012; Paerl & Huisman, 2008; Reynolds, 1984; Sommer, Gliwicz, Lampert, & Duncan, 1986).

Interactions between temperature, nutrients and grazing were idiosyncratic among lakes. Most notably, the interaction between nutrient addition and warming showed opposite effects for net growth rates in the medium and high productivity communities. Although warming stimulated growth for both communities at ambient nutrient levels, warming amplified the effect of nutrient addition in the high productivity community but dampened the nutrient effect in the medium productivity community, despite filamentous cyanobacterial dominance in both communities. This contrasting response might have been caused by the temperature of the warming treatments, which were determined relative to the ambient lake temperature at the time of sampling (see Materials and Methods). The medium productivity community experienced the highest temperatures (20ºC in warmed treatments; see Materials and Methods) and therefore the warming treatment might have surpassed optimal temperatures for growth (Litchman et al., 2010), a condition that can be exacerbated by nutrient addition (Rigosi et al., 2014). Alternatively, these different outcomes could have resulted from enhanced grazing rates of microzooplankton with warming (Chen, Landry, Huang, & Liu, 2012), for which losses could have been compensated in the most productive system but not in the medium productive community.

### 4.4 Warming effects on stoichiometry

The effects of warming on stoichiometry varied by community and size fraction, consistent with the variety of responses reported in previous studies. Although warming decreased C:P and N:P in the larger size fraction of the low productivity community, it increased N:P in the smaller size fraction of the same community. Moreover, warming also caused an increase in the C:P and N:P of the smaller size fraction of the high productivity community (Figures 3a and 4a). Earlier studies have reported various effects of warming on seston stoichiometry. For instance, warming reduced seston C:P and N:P in phytoplankton communities during a spring to summer period, likely as a result of nutrient recycling by heterotrophic microbes (Velthuis et al., 2017). In contrast, warming has also been shown to increase phytoplankton C:P ratios, but only under nutrient‐limiting conditions, possibly resulting from enhanced P use efficiencies (De Senerpont Domis et al., 2014; Verbeek, Gall, Hillebrand, & Streibel, 2018). Warming may also cause an increase in N:P as a result of changes in elemental resource allocation during protein synthesis (Toseland et al., 2013). For the high productivity lake, DIN was significantly lower in all warming treatments, whereas DIP concentrations were the same across all treatments (Figure S1, Table S1). This suggests that N uptake might have increased relative to P in warmed treatments, causing N:P to increase (Figure 3). This pattern is consistent with the finding that warming caused eukaryotic phytoplankton to increase rates of protein synthesis while reducing the density of P‐rich ribosomes necessary to produce cellular proteins, resulting in higher N demand and higher N:P (Toseland et al., 2013).

Interactions between warming and nutrient addition also altered stoichiometry, which furthermore depended on community size structure and composition. In the larger size fraction of the low productivity community, nutrient addition dampened the reduction of N:P due to warming, and a similar dampening effect was observed in the larger size fraction of the medium productivity community, where nutrient addition reversed a reduction in C:P in warm, grazer addition treatments (Figures 3 and 4). This is consistent with earlier reports showing stronger effects of temperature on stoichiometry at low rather than high nutrient loads (De Senerpont‐Domis et al., 2014; Verbeek et al., 2018). It is conceivable that when nutrients are in ample supply, enhanced metabolic rates from warming can be invested in growth, leading to enhanced biomass buildup. Under nutrient limitation, however, growth is constrained and elements may instead accumulate in the cell, leading to stoichiometric shifts. Comparable interactive effects of nutrients have been reported for elevated *p*CO_2_, which caused an increase in cyanobacterial biomass without a change in stoichiometry when nutrients were available in excess, but caused an increase in cyanobacterial C:N ratios without a change in biomass when N was limiting (Verspagen, Van de Waal, Finke, Visser, & Huisman, 2014). Our findings suggest that, similar to the effect of elevated *p*CO_2_, warming may lead to higher phytoplankton (particularly cyanobacterial) biomass when nutrients are available in excess, but may cause shifts toward higher C:nutrient ratios when nutrients are limiting.

### 4.5 Grazing effects on growth and stoichiometry

Grazing had the greatest effect on all measured response variables in the lowest productivity community. Grazing significantly reduced net growth rates in the low productivity community, likely because it had the highest proportion of cells in the edible smaller size fraction (i.e. <30 µm; Figure 1). This result is consistent with the expectation that small cells dominate low nutrient environments but are also more susceptible to grazers (Grover, 1995; Leibold, 1989, 1996; reviewed in Litchman & Klausmeier, 2008). Moreover, for the low productivity community, C:P ratios of the smaller size fraction and N:P of the larger size fraction were reduced by grazing at ambient nutrient levels, suggesting that cells in the low productivity lake can effectively take up recycled P from grazing (Figures 3 and 4; Elser & Urabe, 1999). Grazing effects were less apparent in the medium and high productivity communities, which were dominated by filamentous cyanobacteria that are largely inedible and presumed to be of poor nutritional quality to zooplankton (Frenken et al., 2018; Urabe & Waki, 2009). Although not a typical grazer of the pelagic zone in lakes, we used *D. magna* because it can be cultured with ease and is a generalist grazer that allowed us to standardize grazing pressure in our experiments. It is unlikely that our results were strongly biased by the ability of *D. magna* to ingest larger food particles (0.6–40 µm, Geller & Muller, 1981) as compared to smaller sized *Daphnia*, because the filamentous cyanobacteria in our experiments have been shown to be inedible even by *D. magna* (DeMott, Gulati, & Van Donk, 2001). This is also supported by our observation that *Daphnia* biomass at the end of the experiment was significantly lower when exposed to the high productivity communities dominated by filamentous cyanobacteria as compared to the low productivity community.

## 5 CONCLUSIONS

Our results indicate that climate warming, nutrient enrichment and grazing elicit distinct responses in lake phytoplankton communities depending on the trophic state, community composition and size structure. Across a gradient of increasing productivity, we show that the fraction of small cells in communities decreases, resulting in a decreasing influence of grazing and consumer‐driven nutrient recycling on C:P and N:P. Additionally, stoichiometric responses differed by size fraction for all three communities, indicating that traits associated with cell size will mediate community stoichiometry in response to various stressors. The variable effect of warming and its interactions with nutrient addition in each community across our productivity gradient indicate that global trends toward warming temperatures and eutrophication of lake waters are likely to exert distinct and interactive effects on phytoplankton stoichiometry depending on local environmental conditions. Integrating ecological stoichiometry with size‐related traits may help in assessing mechanisms underlying the impacts of global environmental change on phytoplankton communities.
