# Supplementary material for: Phytoplankton growth and stoichiometric responses to warming, nutrient addition and grazing depend on lake productivity and cell size
Source: Glob Chang Biol. 2019 Jun 1;25(8):2751–62. doi: 10.1111/gcb.14660 (PMC6852242; doi:10.1111/gcb.14660)
Supplement: Supplementary file 1 [file GCB-25-2751-s001.docx]

**Supplemental Information**

**Table S1**: P-values from generalized linear models

| **Growth rate** | |  | |  |  |  |  |  |  |
| --- | --- | --- | --- | --- | --- | --- | --- | --- | --- |
| productivity | temp | | nutrients | | grazing | temp*nut | temp*grz | nut*grz | temp*nut*grz |
| Low | 0.30 | | **<0.001***** | | **<0.001***** | 0.061 | 0.52 | 0.83 | 0.91 |
| Medium | 0.42 | | **<0.001***** | | 0.94 | **<0.05*** | 0.35 | 0.32 | 0.49 |
| High | **<0.001***** | | **<0.001***** | | 0.36 | **<0.05*** | 0.25 | 0.19 | 0.36 |
| \| **ln(N:P <30um)** \| \|  \| \|  \|  \|  \|  \|  \|  \| \| --- \| --- \| --- \| --- \| --- \| --- \| --- \| --- \| --- \| --- \| \| productivity \| temp \| \| nutrients \| \| grazing \| temp*nut \| temp*grz \| nut*grz \| temp*nut*grz \| \| Low \| **<0.01**** \| \| **<0.001***** \| \| 0.37 \| 0.40 \| 0.60 \| 0.25 \| 0.22 \| \| Medium \| 0.74 \| \| **<0.001***** \| \| 0.10 \| **<0.05*** \| 0.16 \| 0.22 \| 0.96 \| \| High \| **<0.05*** \| \| **<0.001***** \| \| 0.23 \| 0.40 \| 0.42 \| 0.23 \| 0.16 \| \| **ln(N:P >30um)** \| \|  \| \|  \|  \|  \|  \|  \|  \| \| productivity \| temp \| \| nutrients \| \| grazing \| temp*nut \| temp*grz \| nut*grz \| temp*nut*grz \| \| Low \| **<0.001***** \| \| **<0.001***** \| \| **<0.001***** \| **<0.05*** \| 0.32 \| 0.090 \| 0.23 \| \| Medium \| 0.44 \| \| **<0.001***** \| \| 0.15 \| 0.42 \| 0.98 \| 0.32 \| 0.055 \| \| High \| 0.42 \| \| **<0.001***** \| \| 0.77 \| 0.51 \| 0.57 \| 0.71 \| 0.50 \| \| **ln(C:P <30um)** \| \|  \| \|  \|  \|  \|  \|  \|  \| \| productivity \| temp \| \| nutrients \| \| grazing \| temp*nut \| temp*grz \| nut*grz \| temp*nut*grz \| \| Low \| 0.25 \| \| **<0.001***** \| \| **<0.001***** \| 0.87 \| 0.51 \| **<0.001***** \| 0.11 \| \| Medium \| 0.34 \| \| **<0.001***** \| \| 0.37 \| 0.44 \| 0.99 \| 0.055 \| 0.56 \| \| High \| **<0.001***** \| \| **<0.001***** \| \| 0.14 \| 0.83 \| 0.39 \| 0.10 \| 0.085 \| \| **ln(C:P >30um)** \| \|  \| \|  \|  \|  \|  \|  \|  \| \| productivity \| temp \| \| nutrients \| \| grazing \| temp*nut \| temp*grz \| nut*grz \| temp*nut*grz \| \| Low \| **<0.001**** \| \| **<0.001***** \| \| 0.70 \| 0.26 \| 0.83 \| 0.89 \| 0.34 \| \| Medium \| 0.59 \| \| **<0.001***** \| \| 0.16 \| 0.65 \| 0.75 \| 0.28 \| **<0.05*** \| \| High \| 0.24 \| \| **<0.001***** \| \| 0.78 \| 0.61 \| 0.61 \| 0.71 \| 0.62 \| | | | | | | | | | |
| **ln(DIN (uM))** | |  | |  |  |  |  |  |  |
| productivity | temp | | nutrients | | grazing | temp*nut | temp*grz | nut*grz | temp*nut*grz |
| Low | 0.38 | | **<0.001***** | | **<0.001***** | 0.87 | 0.14 | 0.32 | **<0.05*** |
| Medium | 0.36 | | **<0.001***** | | 0.53 | 0.08 | 0.87 | 0.14 | 0.51 |
| High | **<0.001***** | | **<0.001***** | | 0.53 | **<0.05*** | 0.23 | 0.24 | 0.22 |
| **ln(DIP (uM))** | |  | |  |  |  |  |  |  |
| productivity | temp | | nutrients | | grazing | temp*nut | temp*grz | nut*grz | temp*nut*grz |
| Low | 0.90 | | **<0.001***** | | **<0.001***** | 0.84 | 0.55 | **<0.001***** | 0.60 |
| Medium | **<0.05*** | | **<0.05*** | | 0.42 | **<0.05*** | 0.42 | 0.42 | 0.42 |
| High | 0.30 | | 0.76 | | 0.29 | 0.46 | 0.61 | 1.00 | 0.78 |

**Figure S1:** Dissolved inorganic nutrients in experimental treatments across a productivity gradient with (a) mean dissolved inorganic nitrogen (DIN) values ±SE (n=4) for each combination of temperature, grazing and nutrient treatments and (b) mean dissolved inorganic phosphorus (DIP) values ±SE (n=4) for each combination of temperature, grazing and nutrient treatments).
