# Supplementary figures and images for: Phytoplankton growth and stoichiometric responses to warming, nutrient addition and grazing depend on lake productivity and cell size
Source: Glob Chang Biol. 2019 Jun 1;25(8):2751–62. doi: 10.1111/gcb.14660 (PMC6852242; doi:10.1111/gcb.14660)

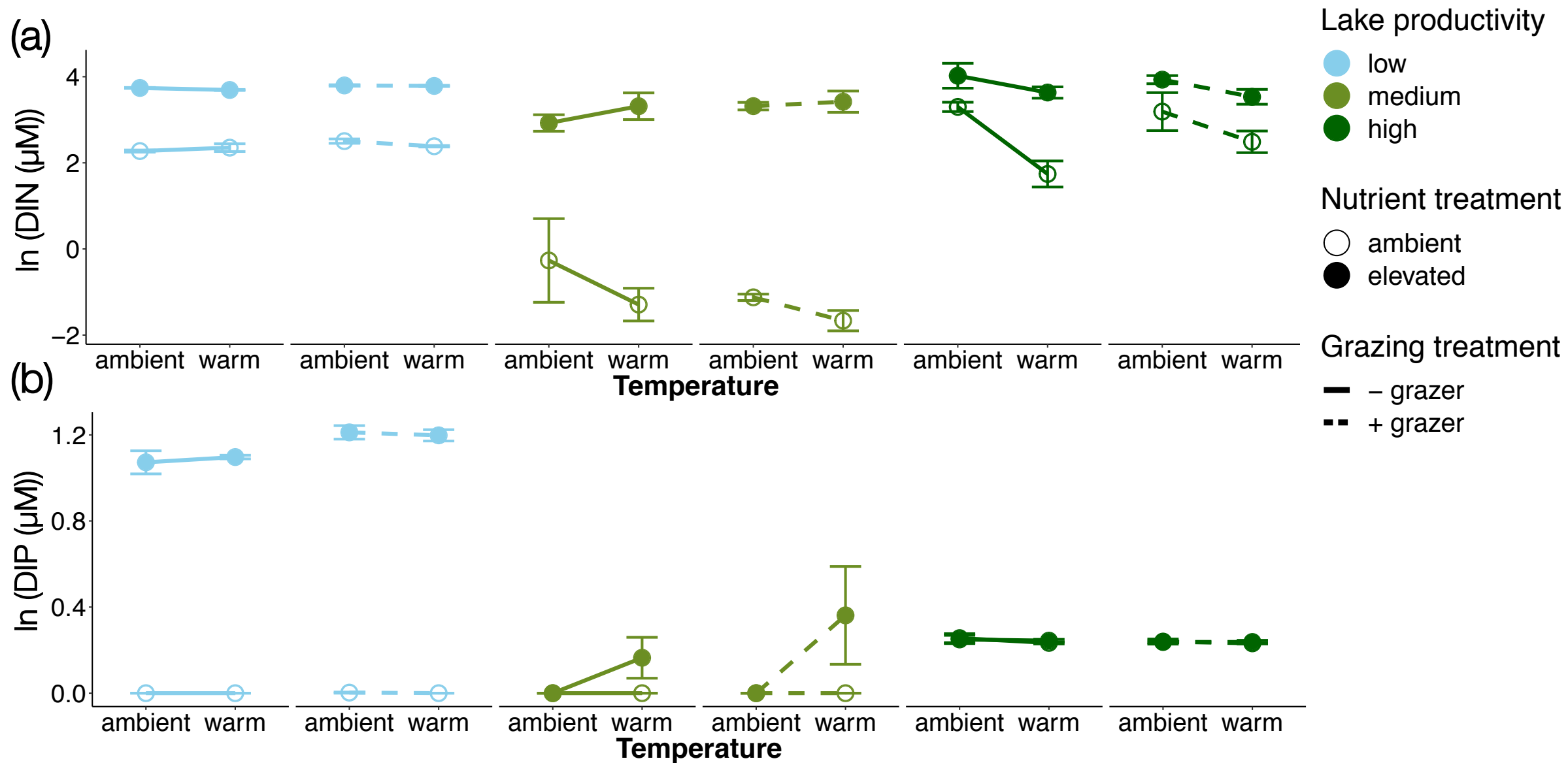

Supplement: Supplementary file 2 [file GCB-25-2751-s002.pdf]
